# Clinical Efficacy of Extracorporeal Cardiopulmonary Resuscitation for Adults with Cardiac Arrest: Meta-Analysis with Trial Sequential Analysis

**DOI:** 10.1155/2019/6414673

**Published:** 2019-07-09

**Authors:** Zhen Chen, Changzhi Liu, Jiequn Huang, Peiling Zeng, Jingcheng Lin, Ruiqiu Zhu, Jianhai Lu, Zhujiang Zhou, Liuer Zuo, Genglong Liu

**Affiliations:** ^1^Intensive Care Unit, Shunde Hospital, Southern Medical University (the First People's Hospital of Shunde), Foshan 528308, Guangdong Province, China; ^2^Department of Pathology, Affiliated Cancer Hospital and Institute of Guangzhou Medical University, Guangzhou, 510095, Guangdong Province, China

## Abstract

**Objective:**

This meta-analysis with trial sequential analysis (TSA) compared the clinical efficacy of extracorporeal cardiopulmonary resuscitation (ECPR) with conventional CPR (CCPR) for adult patients who experienced in-hospital cardiac arrest (IHCA) or out-of-hospital CA (OHCA).

**Methods:**

A literature search was used to identify eligible publications (up to 30 July 2018) from PubMed, the Cochrane Library, the ISI Web of Knowledge, and Embase. Two investigators independently conducted the literature search, study selection, data extraction, and quality evaluation. Meta-analysis and TSA were used to analyze each outcome, and the Grading of Recommendations Assessment, Development, and Evaluation (GRADE) was used to evaluate the level of evidence. The primary outcome was 30-day survival, and the secondary outcomes were 30-day neurologic outcome, 3-6 months' survival, 3-6 months' neurological outcome, 1-year survival, and 1-year neurological outcome.

**Results:**

We identified 13 eligible observational studies for the final analysis. Pooled analyses showed that ECPR was associated with a significantly better 30-day survival (RR = 1.60, 95% CI = 1.25–2.06) and 30-day neurologic outcome (RR = 2.69, 95% CI = 1.63–4.46), and TSA confirmed these results. However, subgroup analysis of patients with OHCA indicated that ECPR and CCPR had similar effects on 30-day survival (RR = 1.18, 95% CI = 0.71–1.97), which was not confirmed by TSA. Analysis of OHCA patients indicated that ECPR provided a better 30-day neurological outcome (RR = 3.93, 95% CI = 1.00–15.50), but TSA did not support these results. Analysis of IHCA patients indicated that ECPR was associated with a better 30-day survival (RR 1.90, 95% CI 1.43–2.52) and 30-day neurologic outcome (RR 2.02, 95% CI 1.21–3.39), and TSA supported these results. Other subgroup analyses showed that the results were generally consistent, regardless of nation, propensity score matching, presumed etiology, whether the CA was witnessed or not, and study quality.

**Conclusions:**

Relative to CCPR, ECPR improved the survival and neurological outcome of patients who had IHCA. Compared to IHCA patients, TSA could not confirm better survival and neurologic outcome of ECPR in OHCA patients, suggesting that further studies are needed.

**Trial Registration:**

This trial was registered with PROSPERO (CRD42018100513) on 17 July 2018.

## 1. Introduction

Cardiac arrest (CA) is one of the most common causes of death worldwide, and more than 500,000 adults per year die following out-of-hospital CA (OHCA) or in-hospital CA (IHCA) [1]. Although the survival time of adults with CA has improved over the past two decades, IHCA has a survival rate of only 22% and OHCA has a survival rate below 10% [2, 3]. Noteworthily, despite standardizing basic cardiac resuscitation and postarrest care ns application with individualized therapies, less than 10% of survivors have good neurological outcomes when they were discharged from hospital [4, 5].

Venoarterial extracorporeal membrane oxygenation (VA-ECMO) is an effective adjunct treatment for CA. In the recent years, the use of ECMO in resuscitative medicine is increasing, and general ECMO practice has been summarized in several trials and reviews [6, 7]. Considering that application of extracorporeal CPR (EPCR) maintains organ perfusion and provides time to reverse the cause of CA, apply a left ventricular assist device or heart transplant, or both. The latest recent international guidelines recommend that ECPR, under specific conditions, is used as a rescue therapy for treatment of refractory CA to support the body's circulation in the absence of an adequately functioning cardiac pump, to ensure adequate systemic blood flow, and to prevent end-organ failure [8, 9].

However, the benefits of applying ECPR are not clear and optimal patient selection is not well-understood [10]. Furthermore, the ethical considerations related to using and studying ECPR are complex [11]. Given the recent increase in the availability and usage of ECPR for CA [12], there is a need for a review of the evidence to provide a clearer understanding of the ECPR for CA. Therefore, we performed an updated meta-analysis of observational studies, addressing whether ECPR, compared with CCPR, can improve survival outcome and lead to good neurological outcome in adult patients with CA according to time interval after CA (30 days, 3-6 months, and 1 year). In addition, we analyzed outcomes of subgroups according to location of arrest (OHCA and IHCA), nation, propensity score matching, presumed etiology, whether the CA was witnessed or not, and study quality.

## 2. Methods

This meta-analysis was conducted and reported according to the Preferred Reporting Items for Systematic Reviews and Meta-analyses Statement (PRISMA) [13]. The review protocol was registered at the PROSPERO registry of systematic reviews in July 2018 (registry number: CRD42018100513).

### 2.1. Data Sources

Publications were identified from systematic searches of PubMed, Cochrane Library, ISI Web of Knowledge, and Embase from inception to July 2018. A basic search was performed using the following keywords: (“out-of-hospital cardiac arrest” OR “in-hospital cardiac arrest” OR “cardiac arrest” OR “heart arrest” OR “OHCA” OR “IHCA”) AND (“extracorporeal membrane oxygenation” OR “extracorporeal oxygenation” OR “extracorporeal circulation” OR “extracorporeal life support” OR “percutaneous cardiopulmonary support” OR “ECMO” OR “ECPR” OR “E-CPR” OR “ECLS” OR “PCPS”) AND (“conventional cardiopulmonary resuscitation” OR “cardiopulmonary resuscitation” OR “resuscitation” OR “cardiac massage” OR “heart massage” OR “CCPR” OR “CPR”). No language restrictions were applied for article selection. Additional studies were identified by review of the reference lists of relevant articles.

### 2.2. Eligibility Criteria

All eligible studies examined adult patients (age*⩾*18 years) who had IHCA or OHCA and received ECPR (intervention) or CCPR (control) and reported survival or neurological outcome. The eligibility for inclusion was independently evaluated by two reviewers; in cases of disagreement, a consensus was reached by consultation with a third reviewer. Publications were excluded if they were review articles, correspondences, editorials, meeting abstracts, expert opinions, noncomparative studies, or pediatric studies or if there was insufficient information for data extraction.

### 2.3. Data Extraction

Two reviewers independently extracted the study characteristics and data from each eligible publication, including the first author's name, year of publication, country of origin, study design, location of arrest (OHCA or IHCA), number of patients, sex ratio, mean age, percentage of acute myocardial infarctions (AMIs), rate of return to spontaneous circulation (ROSC), CPR duration, time-to-extracorporeal membrane oxygenation (ECOM), mortality, inclusion criteria, and use of ECPR. The primary outcome measure was 30-day survival. The secondary outcomes were 30-day neurologic outcome, 3-6 months' survival, 3-6 months' neurological outcome, 1-year survival, and 1-year neurological outcome. If 30-day outcome data were not reported, in-hospital outcome data were used. Neurological status was considered favorable when the Pittsburgh Cerebral Performance Category (CPC) score was 1 or 2 or when the Modified Glasgow Outcome Score (MGOS) was 4 or less.

### 2.4. Quality Assessment

The quality of each study was assessed using a modified version of the Newcastle-Ottawa Quality Assessment Scale for Cohort Studies (NOS) [14]. Notably, we added Utstein style which can comprehensively assess whether data reported is sufficient or not for resuscitation trials, to outcome section of NOS. This scale considers ten items, related to selection, comparability, and outcome to evaluate the quality of observational studies and assigns each study a score from 0 to 10 stars. Observational cohort studies with 7 or more stars were considered to be of high quality. The assessment was performed independently by two reviewers, and disagreements were resolved by discussion.

### 2.5. Statistical Analyses

RevMan 5.3 software from the Cochrane Collaboration was utilized for the meta-analysis. Continuous variables were reported as mean (± standard deviation) or median (interquartile range). Categorical variables were expressed as number and percentage (%). For dichotomous outcomes (survival outcome and favorable neurologic outcome), the results are expressed as relative risk (RR) and 95% confidence interval (CI). Given all included studies are observational studies that are heterogeneous by design, data were pooled using Mantel–Haenszel method random-effects weighting.

Heterogeneity in a meta-analysis indicates variability of results among included studies. Heterogeneity was assessed using the Q test,* p *value, and* I*^*2*^ index, with three classes of heterogeneity: low (*I*^*2*^ < 50%), moderate (50% <* I*^*2*^ <75%), and high (*I*^*2*^ > 75%).

The effects of different clinical characteristics of patients on the association of ECPR with survival and neurologic outcome were determined. In this analysis, subgroups were examined based on location of arrest (OHCA* vs.* IHCA* vs.* OHCA and IHCA), nation (eastern countries vs. western countries), propensity score matching (yes* vs.* no), presumed etiology (cardiac* vs.* not stated), witnessed arrest (yes* vs.* not stated), and study quality (high* vs.* low). This analysis determined whether any of the differences between subgroups were statistically significant. We used *χ*2 to test for subgroup differences—that is, whether the observed differences in the subgroups are compatible with chance alone. A low **P** value (or a large *χ*2 statistic relative to its degree of freedom) provides evidence of heterogeneity beyond chance. Publication bias was assessed by examination of funnel plots, when 10 or more trials reported the primary outcomes [15].

### 2.6. Grading the Quality of Evidence

Two investigators independently assessed the quality of evidence for outcomes using the Grading of Recommendations Assessment, Development, and Evaluation (GRADE) and classified each outcome as having high, moderate, low, or very low quality of evidence. These judgments were based on risk of bias, inconsistency, indirectness, imprecision, and publication bias. GRADE Pro-version 3.6 software was used for these analyses.

### 2.7. Trial Sequential Analysis

Trial sequential analysis (TSA) [16] combines* a priori* information on size calculation for a meta-analysis, with the adaptation of monitoring boundaries to evaluate the accumulated evidence [17]. When the cumulative Z-curve crosses the trial sequential monitoring boundary or enters the futility area, this indicates that there is sufficient evidence for the anticipated intervention effect and that no further trials are needed. If the Z-curve does not cross either of the boundaries and the required information size has not been reached, this indicates that there is insufficient evidence to reach a conclusion and that more trials are needed to confirm the results. Information size was calculated as a diversity-adjusted required information size, as suggested by the diversity of the intervention effect estimates among the included trials [18].

For the TSA, the required information size was calculated based on 20% increase in outcomes. The type I error (*α*) was set at 0.05 or 0.01 and the power (1 – *β*) at 0.80. The control event rates were calculated from the CCPR group. The TSA was conducted using TSA version 0.9 beta software (http://www.ctu.dk/tsa) [19].

## 3. Results

### 3.1. Literature Search

**Figure 1:**
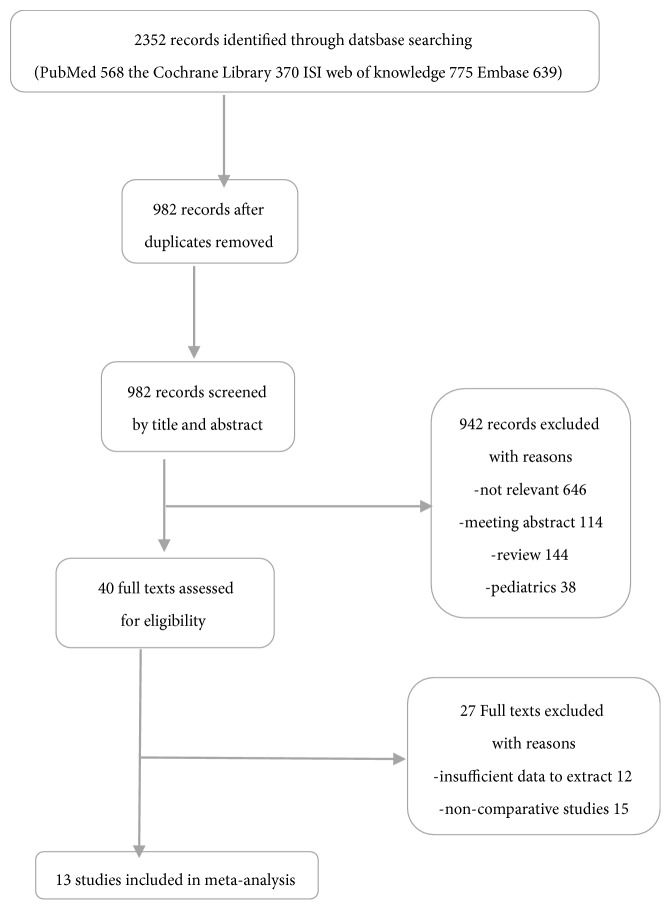
Flow diagram of literature search and selection process of the studies.

Figure 1 shows the study selection procedure. Our initial search identified 568 records from PubMed, 370 from the Cochrane Library, 775 from the ISI Web of Knowledge, and 639 from Embase. We first removed 1370 duplicates and then eliminated 982 papers following inspection of the titles and abstracts. We read the full text of each of the remaining 40 articles. Finally, we identified 13 studies [12, 20–31] that fulfilled our eligibility criteria.

### 3.2. Study and Patient Characteristics

Table 1 shows the main characteristics of the 13 included studies, all of which were observational. These studies were conducted in Korea (n = 5), Taiwan (n = 4), Japan (n = 2), Austria (n = 1), and Germany (n = 1). Eight studies used propensity score matching [20–22, 24, 26, 27, 29, 30]. In addition, 6 studies examined patients with IHCA [20, 21, 23, 26, 29, 30], 5 studies examined patients with OHCA [12, 22, 24, 27, 28], and 2 examinedtrials with IHCA and OHCA [25, 31]. The sample sizes of the studies ranged from 48 to 955, the percentage of males ranged from 56.7% to 89.6%, and the mean age of the patients ranged from 46 to 73 years. Overall, ECPR patients were more likely to suffer from AMI, experience a return of spontaneous circulation (ROSC), and have a longer duration of CPR. Table S1 summarizes the inclusion criteria of study populations and the indications used for administration of ECPR.

### 3.3. Results of the Quality Assessment

Supplementary Table S2 shows the quality assessment of the studies based on the NOS. These results show that 7 studies scored between 7 and 9 points, 2 studies scored 6 points, and the other 4 studies scored below 6 points.

### 3.4. Primary Outcome

**Figure 2:**
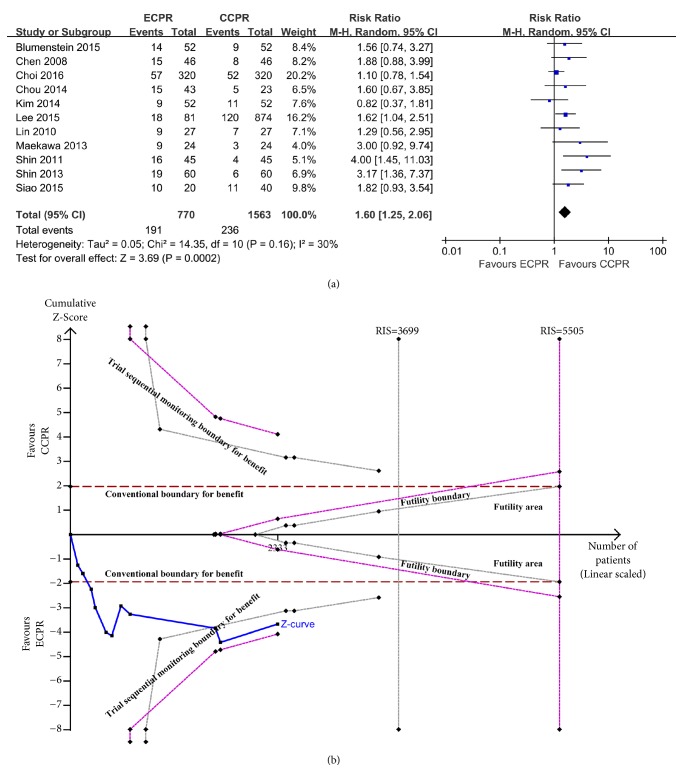
(a) Forest plot of studies reporting 30-day survival outcome. (b) Random-effect model of trial sequential analysis for 30-day survival outcome. Type 1 error is =5%; A diversity-adjusted information size of 3699 participants calculated on the basis of a survival rate of 17.5% in the CCPR group, 20% increase in outcome, *α* = 5% (two sided), *β* = 20%, and **I**^2^ = 30%. Complete blue line represents cumulative Z-curve, which crossed conventional boundary (dashed red line) and the trial sequential monitoring boundary (dashed gray line).

Figure 2(a) shows 30-day survival rate of patients with CA arrest. The usage of ECPR was associated with increased survival (RR = 1.60, 95% CI = 1.25–2.06,* I*^2^ = 30%) compared to CCPR. The TSA with the random-effects model results show that the cumulative Z-curve crossed the conventional boundary and the trial sequential monitoring boundary (Figure 2(b)). These results indicate that this evidence is sufficient and conclusive and that further trials are not required.

### 3.5. Secondary Outcomes

**Figure 3:**
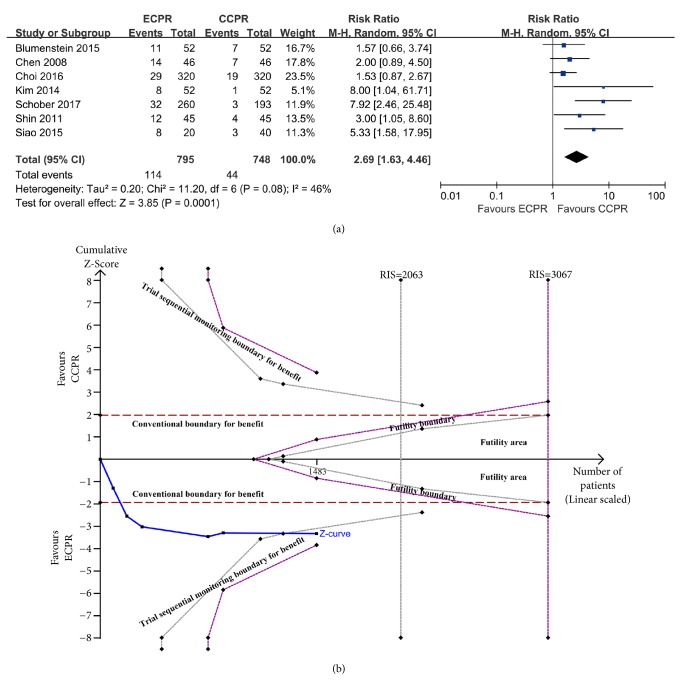
(a) Forest plot of studies reporting 30-day favorable neurologic outcome. (b) Random-effect model of trial sequential analysis for 30-day favorable neurologic outcome. Type 1 error is =5%. A diversity-adjusted information size of 2063 participants calculated on the basis of a good neurologic outcome rate of 7.8% in the CCPR group, 20% increase in outcome, *α* = 5% (two sided), *β* = 20%, and **I**^2^ = 46%. Complete blue line represents cumulative Z-curve, which crossed conventional boundary (dashed red line) and the trial sequential monitoring (dashed gray line).

Our data also indicated that the 30-day good neurologic outcome was better for ECPR than CCPR patients (RR = 2.69, 95% CI = 1.63–4.46,* I*^2^ = 46%) (Figure 3(a)). The TSA, in which the cumulative Z-curve crossed the conventional boundary and the trial sequential monitoring boundary, confirmed these results (Figure 3(b)). Similarly, pooled analyses showed that ECPR was significantly associated with improved survival at 3-6 months (RR = 2.59, 95% CI = 1.71–3.93,* I*^2^ = 0) (Supplementary Figure S1A), neurological outcome at 3-6 months (RR = 4.21, 95% CI 2.47–7.16,* I*^2^ = 0) (Supplementary Figure S2A), survival at 1 year (RR = 1.86, 95% CI 1.29–2.68,* I*^2^ = 0) (Supplementary Figure S3A), and neurological outcome at 1 year (RR = 2.43, 95% CI = 1.48–3.99,* I*^2^ = 0) (Supplementary Figure S4A). Additionally, analysis of the secondary outcomes using TSA showed that all cumulative Z-curves crossed the conventional boundary and the trial sequential monitoring boundary (Supplementary Figures S1B, S2B, S3B, and S4B), thus indicating the evidence is sufficient and conclusive.

### 3.6. Subgroup Analyses

**Figure 4:**
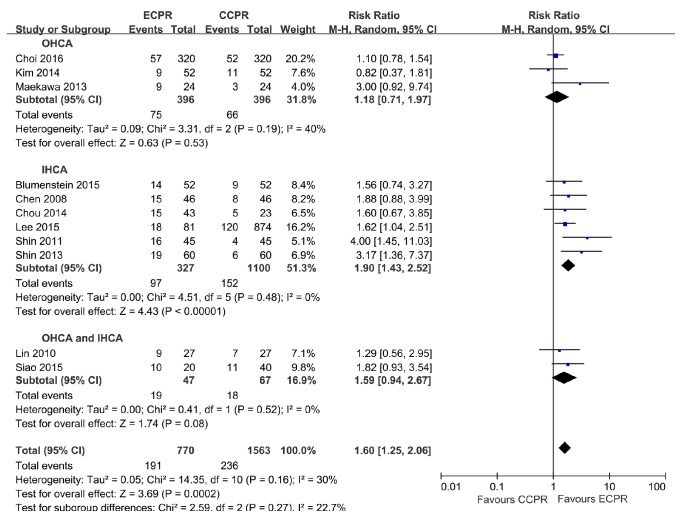
Forest plot of studies with 30-day survival outcome, reporting 30-day survival outcome stratified by location of arrest.

**Figure 5:**
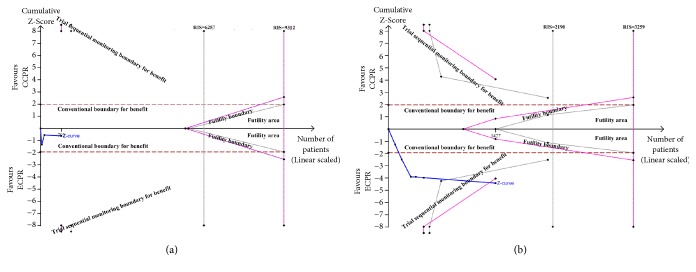
(a) Random-effect model of trial sequential analysis for 30-day survival outcome in OHCA patients. Type 1 error is =5%; a diversity-adjusted information size of 6257 participants calculated on the basis of a survival rate of 16.6% in the CCPR group, 20% increase in outcome, *α* = 5% (two sided), *β* = 20%, and **I**^2^ = 40%. Complete blue line represents cumulative Z-curve, which does not cross the conventional boundary (dashed red line) or the trial sequential monitoring boundary (dashed gray line). (b) Random-effect model of trial sequential analysis for 30-day survival outcome in IHCA patients. Type 1 error is =5%; a diversity-adjusted information size of 2190 participants calculated on the basis of a survival rate of 14.8% in the CCPR group, 20% increase in outcome, *α* = 5% (two sided), *β* = 20%, and **I**^2^ = 0%. Complete blue line represents cumulative Z-curve, which crossed conventional boundary (dashed red line) and the trial sequential monitoring boundary (dashed gray line).

Analysis of patients with OHCA indicated similar 30-day survival for those receiving CCPR and ECPR (RR = 1.18, 95% CI = 0.71–1.97,* I*^***2***^ = 40%) (Figure 4), but the TSA did not confirm these results (Figure 5(a)). Analysis of patients with IHCA indicated that ECPR provided improved 30-day survival (RR = 1.90, 95% CI = 1.43–2.52,* I*^***2***^ = 0) (Figure 4), and the TSA confirmed these results (Figure 5(b)).

**Figure 6:**
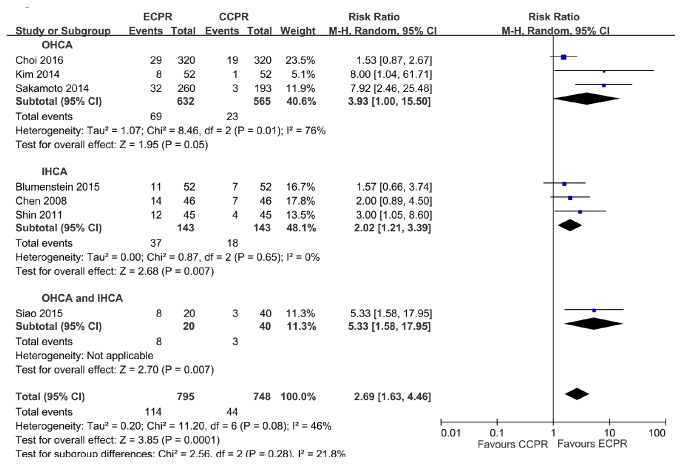
Forest plot of studies with 30-day favorable neurologic outcome, reporting 30-day favorable neurologic outcome stratified by location of arrest.

**Figure 7:**
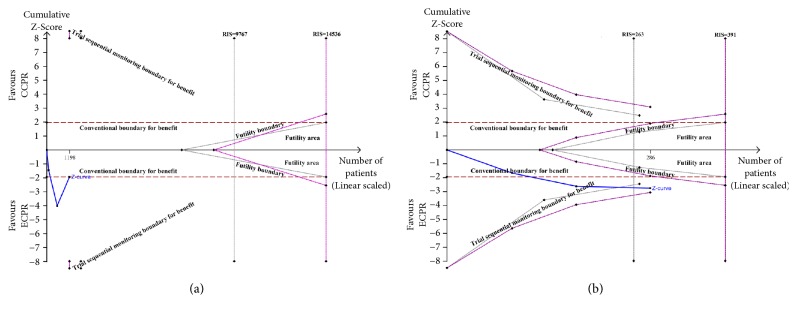
(a) Random-effect model of trial sequential analysis for 30-day favorable neurologic outcome in OHCA patients. Type 1 error is =5%; a diversity-adjusted information size of 9767 participants calculated on the basis of a good neurologic outcome rate of 3.1% in the CCPR group, 20% increase in outcome, *α* = 5% (two sided), *β* = 20%, and **I**^2^ = 76%. Complete blue line represents cumulative Z-curve, which just reached the conventional boundary (dashed red line) and did not cross the trial sequential monitoring boundary (dashed gray line). (b) Random-effect model of trial sequential analysis for 30-day favorable neurologic outcome in IHCA patients. Type 1 error is =5%; a diversity-adjusted information size of 263 participants calculated on the basis of a good neurologic outcome rate of 12.5% in the CCPR group, 20% increase in outcome, *α* = 5% (two sided), *β* = 20%, and **I**^2^ = 0%. Complete blue line represents cumulative Z-curve, which crossed conventional boundary (dashed red line) and the trial sequential monitoring boundary (dashed gray line).

Analysis of patients with OHCA indicated ECPR provided better 30-day neurologic outcome than CCPR (RR = 3.93, 95% CI = 1.00–15.50,* I*^***2***^ = 76%) (Figure 6), but the TSA results showed that the cumulative Z-curve just reached the conventional boundary, and did not cross the trial sequential monitoring boundary (Figure 7(a)). Analysis of patients with IHCA indicated that ECPR provided better 30-day neurologic outcome than CCPR (RR = 2.02, 95% CI = 1.21–3.39,* I*^***2***^ = 0) (Figure 6), and the TSA confirmed these results (Figure 7(b)).

ECPR and CCPR provided similar 30-day survival rates for OHCA and IHCA patients (OR = 1.59, 95% CI = 0.94–2.67,* I*^***2***^ = 0). Only 1 trial with OHCA and IHCA patients reported 30-day neurologic outcome, so data pooled was impossible. Other subgroup analyses showed significant differences within subgroups based on nation (only 1 trail with western countries reported 30-day survival outcome, so data pooled was impossible), propensity score matching, presumed etiology, witnessed arrest, and study quality (Table 2).

### 3.7. Publication Bias

Assessment of potential publication bias for the primary outcome (30-day survival) showed there was no bias among the included trials, as indicated by the presence of all results within the “funnel” (Supplementary Figure S5).

### 3.8. GRADE

The GRADE level of evidence was moderate for 30-day favorable neurologic outcome in IHCA patients and for 3-6 months' survival, 3-6 months' favorable neurological outcome, and 1 year favorable neurological outcome. The GRADE level of evidence was low for 30-day survival in IHCA patients and for 30-day survival, 30-day favorable neurologic outcome, and 1 year survival outcome. The GRADE level of evidence was very low for 30-day survival and 30-day favorable neurologic outcome in OHCA patients. Supplementary Table S3 shows the GRADE evidence profiles. The main reasons for a deceased grade were the use of an observational cohort design, inconsistency, and imprecision. However, due to the large magnitude of the effect sizes (RR > 2), the grade increased by +1.

## 4. Discussion

Our systematic review and meta-analysis, which compared ECPR and CCPR for patients with CA, showed that ECPR was associated with an improved 30-day survival (RR = 1.60, 95% CI = 1.25–2.06) and an improved 30-day neurologic outcome (RR = 2.69, 95% CI = 1.63–4.46) of patients with IHCA. However, ECPR had no effect on the survival or neurologic outcome of patients with OHCA.

Several previous meta-analyses have also compared ECPR and CCPR [32–35], but there are several important differences between the present and these previous meta-analyses. First, our meta-analysis included one additional trial that was not included in the previous meta-analyses. Second, we registered the protocol of this study on PROSPERO to increase the transparency and quality of the meta-analysis. Third, we used TSA (a more conservative approach) to confirm the conclusions and estimate the required information size. Finally, we analyzed the level of evidence using GRADE, which classifies the conclusions of studies as high, moderate, low, or very low quality of evidence.

Our analysis of survival and neurologic outcome among all 13 studies showed that ECPR provided a beneficial effect at 30 days, 3 to 6 months, and 1 year after CA, and the TSA results supported these conclusions. Although several of the 13 trials used propensity-score matching, there were still differences in the baseline characteristics of patients receiving ECPR and CCPR. In particular, ECPR-treated patients were more likely to be male, younger, and suffering from AMI, all of which are associated with increased survival in this setting [36, 37]. Hence, it is difficult to reliably distinguish the effects of ECPR and CCPR because of the bias and confounds that are inherent to included cohort studies. In additional, absence of randomized controlled trials (RCTs) and all eligible studies were observational cohort studies. Analysis using GRADE indicated that the certainty of the body of evidence was low for a benefit from ECPR. These results were similar to latest results of the International Liaison Committee on Resuscitation (ILCOR) systematic review [38].

Subgroup analysis of IHCA patients indicated ECPR was associated with better survival and neurologic outcome than CCPR. Additionally, heterogeneity was low in the studies of IHCA patients, indicating the consistency of these results. We presumed that the association of ECPR with better survival rates may be due to cardiac events in IHCA patients being detected more promptly by clinicians, and ECPR could be selectively implemented in patients deemed to have reversible cardiac arrest causes after application of ECPR [39]. In contrast, subgroup analysis of OHCA patients indicated ECPR provided no benefit in terms of survival. Compared to ECPR, pooled analysis showed better neurologic outcome to CCPR in OHCA patients, which was not verified by TSA. Furthermore, heterogeneity was high among OHCA patients, indicating inconsistency of the results. These results may be explained by the impact of other factors, such as quality of bystander CPR, use of an automated external defibrillator, longer time of no flow/low flow, immediate witness, and prehospital emergency medical system variables [10, 40]. Thus, a number of more variables can affect the outcome of OHCA patients compared to IHCA patients [41].

The detailed indications for ECPR, as well as inclusion criteria of study populations, varied greatly among the 13 studies we examined. Thus, variable and often flexible indications were applied to select patients for ECPR. We conducted other subgroup analyses with stratification by inclusion criteria to identify conditions (*e.g.*, presumed etiology, witnessed CA) that could impact the outcomes of patients receiving ECPR. Subgroup analysis based on presumed etiology and witnessed CA showed significant differences in that patients receiving ECPR had improved survival and neurologic outcome. Additionally, patients with witnessed CA arrest had better survival than those without witnessed CA (RR = 2.22, 95% CI = 1.57–3.14* vs.* RR = 1.26, 95% CI = 1.01–1.59, interaction, **P** = 0.03). It is commonly assumed that patients with witnessed CA receive CPR more promptly and have improved outcome because of the more rapid delivery of prehospital defibrillation and other interventions [42]. A previous analysis of 1195 patients indicated that witnessed CA was associated with a better outcome, ROSC (22.7%), and prolonged survival to hospital discharge (9.7%) [43].

It is worth mentioning that ECPR did not provide survival benefit for patients who had OHCA, but TSA analysis indicated the cumulative Z-curve did not enter the futility boundary. Similarly, analysis of patients with OHCA indicated that ECPR showed better neurologic outcome, but the TSA results showed that the cumulative Z-curve did not cross the trial sequential monitoring boundary. These results indicated that the required information size was not achieved, the evidence needed to reach a conclusion was insufficient, and future large and adequately powered prospective clinical trials were needed to confirm whether patients who had OHCA can obtain benefit from ECPR or not.

Currently, ECPR is a method used to treat refractory cardiac arrest. Available studies are nonrandomized and compare ECPR (a bail-out therapy in refractory arrest) with historical or unmatched groups of patients who received CCPR (treated until ROSC or death), which introduces a huge selection bias: ECPR patients obviously do not achieve ROSC through initial CCPR, thus are sicker and have lower chances to survive, but are compared to those with nonrefractory arrest. Hence, trails comparing early extracorporeal life support (ECLS) (non-bail-out, but early proactive ECLS to precede ROSC) vs. continued CCPR are urgently needed to explore the potential benefit of ECPR in the future.

There were several limitations of this systematic review. First, there were a relatively small number of observational studies and no RCTs, so there was a possibility of unbalanced confounders. In other words, subjective selection of ECPR candidates may have biased the estimate of survival outcomes. Second, only a limited number of studies reported complications from ECPR use. The most frequently reported complications were issues related to cannulation, in particular, significant bleeding and leg ischemia, and ECMO-related complications including accidental decannulation or circuit failure. Identifying and minimizing complications remain a key step in effective and safe use of ECPR. Third, none of the included studies reported scores for the severity of CA. Successful rescue angioplasty guarantees the survival of patients after ROSC. In contrast, patients have a reduced chance of survival after failed rescue angioplasty even after undergoing ECPR. The failure of coronary angioplasty correlates with the number of obstructed coronary vessels and the severity of each obstruction. Patients with triple vessel disease may require heart surgery. Similarly, the severity score is closely associated with survival and neurologic outcome.

## 5. Conclusion

Compared to CCPR, use of ECPR may improve the survival and neurologic outcome of patients with IHCA. In contrast to IHCA patients, TSA could not confirm better survival and neurologic outcome of ECPR in OHCA patients, suggesting that further studies are needed. Additionally, clinicians need to more rigorously define the criteria to be used for selection of patients with CA who will benefit most from ECPR.

## Figures and Tables

**(a):** 

Study(year)	Nation	Study design	OHCA/IHCA	Patients (N.)	Male (%)	Mean age ECPR/CCPR (years)
Blumenstein (2015)	Germany	CS and PSM	IHCA	104	56.7	72/73
Chen (2008)	Taiwan	CS and PSM	IHCA	92	85.9	57/55
Choi (2016)	Korea	CS and PSM	OHCA	640	81	56/58
Chou (2014)	Taiwan	CS	IHCA	66	86.4	60.5/69.6
Kim (2014)	Korea	CS and PSM	OHCA	104	76.5	54/54
Lee (2015)	Korea	CS	IHCA and OHCA	955	64.9	59.0/63.5
Lin (2010)	Taiwan	CS and PSM	IHCA	54	81.5	59/60
Maekawa (2013)	Japan	CS and PSM	OHCA	48	79.2	57/57
Sakatomo (2014)	Japan	CS	OHCA	454	89.6	56.3/58.1
Schober (2017)	Austria	CS	OHCA	239	74	46/60
Shin (2011)	Korea	CS and PSM	IHCA	90	64.7	63.5/61.5
Shin (2013)	Korea	CS and PSM	IHCA	120	64.2	60.8/60.5
Siao (2015)	Taiwan	CS	IHCA and OHCA	60	76.7	54.6/60.3

**(b):** 

Study(year)	AMI ECPR/CCPR (%)	ROSC rate ECPR/CCPR (%)	CPR duration ECPR/CCPR (min)	Time-to-ECOM (min)	Mortality (%)
Blumenstein (2015)	28.9/36.6	NR/NR	33(19-47)/ 37(30-45)^#^	NR	77.9%
Chen (2008)	61/72	42/24	53 (41)/47 (33)*∗*	NR	75%
Choi (2016)	NR/NR	NR/NR	35(19-54.5)/28(15-37)^#^	NR	83.3%
Chou (2014)	100/100	100/52.1	NR/NR	59.7±34.1*∗*	69.7%
Kim (2014)	84.6/88.5	30.8/32.7	62.5(49-88)/ 60.5(40-83.5)^#^	68.5 (32-132)^#^	80.8%
Lee (2015)	NR/NR	86.4/73.3	43(21-60)/30(15-48)^#^	NR	85.5%
Lin (2010)	65.5/73.0	100/100	41.8 (19.8)/40.0 (26.4)*∗*	NR	70.4%
Maekawa (2013)	NR/NR	4.2/12.5	49(43-66)/ 52(43-65)^#^	NR	75%
Sakatomo (2014)	63.5/59.3	NR/NR	NR/NR	NR	NR
Schober (2017)	NR/NR	57/38	97(79-147)/ 77(58-95)^#^	55(48-65)^#^	NR
Shin (2011)	48.8/48.8	35/21	37.9(19.3)/36.8(19.3)*∗*	NR	77.8%
Shin (2013)	43.3/35.0	75/48.3	38.8(20.7)/38.1(21.0)*∗*	NR	68.9%
Siao (2015)	60/40	95/47.5	NR/NR	NR	65%

Data are reported as mean ± standard deviation or median (interquartile range); #: median (IQR); *∗*: mean (SD). Abbreviations: OHCA=out-of-hospital cardiac arrest, IHCA=in-hospital cardiac arrest, N=number, NR=not reported, ECPR=extracorporeal cardiopulmonary resuscitation, CCPR=conventional cardiopulmonary resuscitation, AMI= acute myocardial infarction, ROSC=return of spontaneous circulation, CPR= cardiopulmonary resuscitation, CS=cohort study, PSM= propensity score matching, and ECOM= extracorporeal membrane oxygenation.

**Table 2:** Results of subgroup analysis based on different standards.

Characteristics	Survival outcome					Neurologic outcome				
	N	RR(95%CI)	*P* value for heterogeneity	*I* ^2^ (%)	*P* values Between subgroups	N	RR(95%CI)	*P* value for heterogeneity	*I* ^2^(%)	*P* values between subgroups
All	11	1.60(1.25-2.06)	0.16	30		7	2.69(1.63-4.46)	0.08	46	
Nation					0.91					0.21
Eastern countries	10	1.63(1.23-2.13)	0.11	37		5	2.23(1.52-3.27)	0.02	29	
Western countries	1	1.56(0.74-3.27)	NA	NA		2	3.66(1.85-7.25)	0.23	81	
Propensity score matching					0.97					0.008
Yes	8	1.65(1.14-2.40)	0.06	49		5	1.87(1.29-2.71)	0.48	0	
No	3	1.67(1.19-2.34)	0.96	0		2	6.97(2.89-16.79)	0.63	0	
Presumed aetiology					0.31					0.74
Cardiac-origin	6	1.37(1.06-1.76)	0.44	0		4	2.57(1.74-3.82)	0.03	65	
Not stated	5	1.80(1.13-2.89)	0.08	52		3	2.58(1.39-4.82)	0.28	21	
Witnessed arrest					0.03					0.23
Witnessed	6	2.22(1.57-3.14)	0.42	0		3	2.02(1.21-3.39)	0.65	0	
Not stated	5	1.26(1.01-1.59)	0.35	10		4	4.08(1.46-11.37)	0.02	70	
Study quality					0.20					0.43
High quality	7	1.90(1.39-2.59)	0.15	37		4	2.20(1.33-3.62)	0.46	0	
Low quality	4	1.32(1.04-1.68)	0.39	1		3	3.65(1.15-11.54)	0.01	77	

Abbreviations: N= number of participants, NA=not application, RR= relative risk, and CI=confidence interval;

## Abbreviations

TSA: Trial sequential analysis
ECPR: Extracorporeal cardiopulmonary resuscitation
CA: Cardiac arrest
GRADE: Grading of Recommendations Assessment, Development, and Evaluation
RR: Relative risk
OHCA: Out-of-hospital cardiac arrest
IHCA: In-hospital cardiac arrest
ECLS: Extracorporeal life support
CCPR: Conventional cardiopulmonary resuscitation
VA-ECOM: Venoarterial extracorporeal membrane oxygenation
ECOM: Extracorporeal membrane oxygenation
PRISMA: Preferred Reporting Items for Systematic Reviews and Meta-analyses Statement
AMI: Acute myocardial infarction
ROSC: Return of spontaneous circulation
CPR: Cardiopulmonary resuscitation
CPC: Cerebral Performance Category
MGOS: Modified Glasgow Outcome Score
RCTs: Randomized controlled trials
ILCOR: International Liaison Committee on Resuscitation.

## Supplementary Materials

Supplementary 1 Supplementary Table S1: Inclusion criteria of study population and indication of extracorporeal cardiopulmonary resuscitation (ECPR).

Supplementary 2 Supplementary Table S2: Assessment of study quality using a modified version of the Newcastle-Ottawa Quality Assessment Scale for Cohort studies.

Supplementary 3 Supplementary Table S3: Summary of findings table.

Supplementary 4 Supplementary Figure S1: (A) Forest plot of studies reporting 3-6 months' survival outcome. (B) Random-effect model of trial sequential analysis for 3-6 months' survival outcome. Type 1 error is =5%; a diversity-adjusted information size of 1231 participants calculated on the basis of a survival rate of 10.5% in the CCPR group, 20% increase in outcome, *α* = 5% (two sided), *β* = 20%, and **I**^2^ = 0%. Complete blue line represents cumulative Z-curve, which crossed conventional boundary (dashed red line) and the trial sequential monitoring boundary (dashed gray line).

Supplementary 5 Supplementary Figure S2: (A) Forest plot of studies reporting 3-6 months' favorable neurologic outcome. (B) Random-effect model of trial sequential analysis for 3-6 months' favorable neurologic outcome. Type 1 error is =5%; a diversity-adjusted information size of 1445 participants calculated on the basis of a good neurologic outcome rate of 5.0% in the CCPR group, 20% increase in outcome, *α* = 5% (two sided), *β* = 20%, and **I**^2^ = 0%. Complete blue line represents cumulative Z-curve, which crossed conventional boundary (dashed red line) and the trial sequential monitoring boundary (dashed gray line).

Supplementary 6 Supplementary Figure S3: (A) Forest plot of studies reporting 1-year survival outcome. (B) Random-effect model of trial sequential analysis for 1-year survival outcome. Type 1 error is =5%; a diversity-adjusted information size of 660 participants calculated on the basis of a survival rate of 15.4% in the CCPR group, 20% increase in outcome, *α* = 5% (two sided), *β* = 20%, and **I**^2^ = 0%. Complete blue line represents cumulative Z-curve, which crossed conventional boundary (dashed red line) and the trial sequential monitoring boundary (dashed gray line).

Supplementary 7 Supplementary Figure S4: (A) Forest plot of studies reporting 1-year favorable neurologic outcome. (B) Random-effect model of trial sequential analysis for 1-year favorable neurologic outcome. Type 1 error is =5%; a diversity-adjusted information size of 546 participants calculated on the basis of a good neurologic outcome rate of 9.2% in the CCPR group, 20% increase in outcome, *α* = 5% (two sided), *β* = 20%, and **I**^2^ = 0%. Complete blue line represents cumulative Z-curve, which crossed conventional boundary (dashed red line) and the trial sequential monitoring boundary (dashed gray line).

Supplementary 8 Supplementary Figure S5: Assessment of publication bias using a funnel plot.
