# Supplementary figures and images for: Clinical Efficacy of Extracorporeal Cardiopulmonary Resuscitation for Adults with Cardiac Arrest: Meta-Analysis with Trial Sequential Analysis
Source: Biomed Res Int. 2019 Jul 9;2019:6414673. doi: 10.1155/2019/6414673 (PMC6652040; doi:10.1155/2019/6414673)

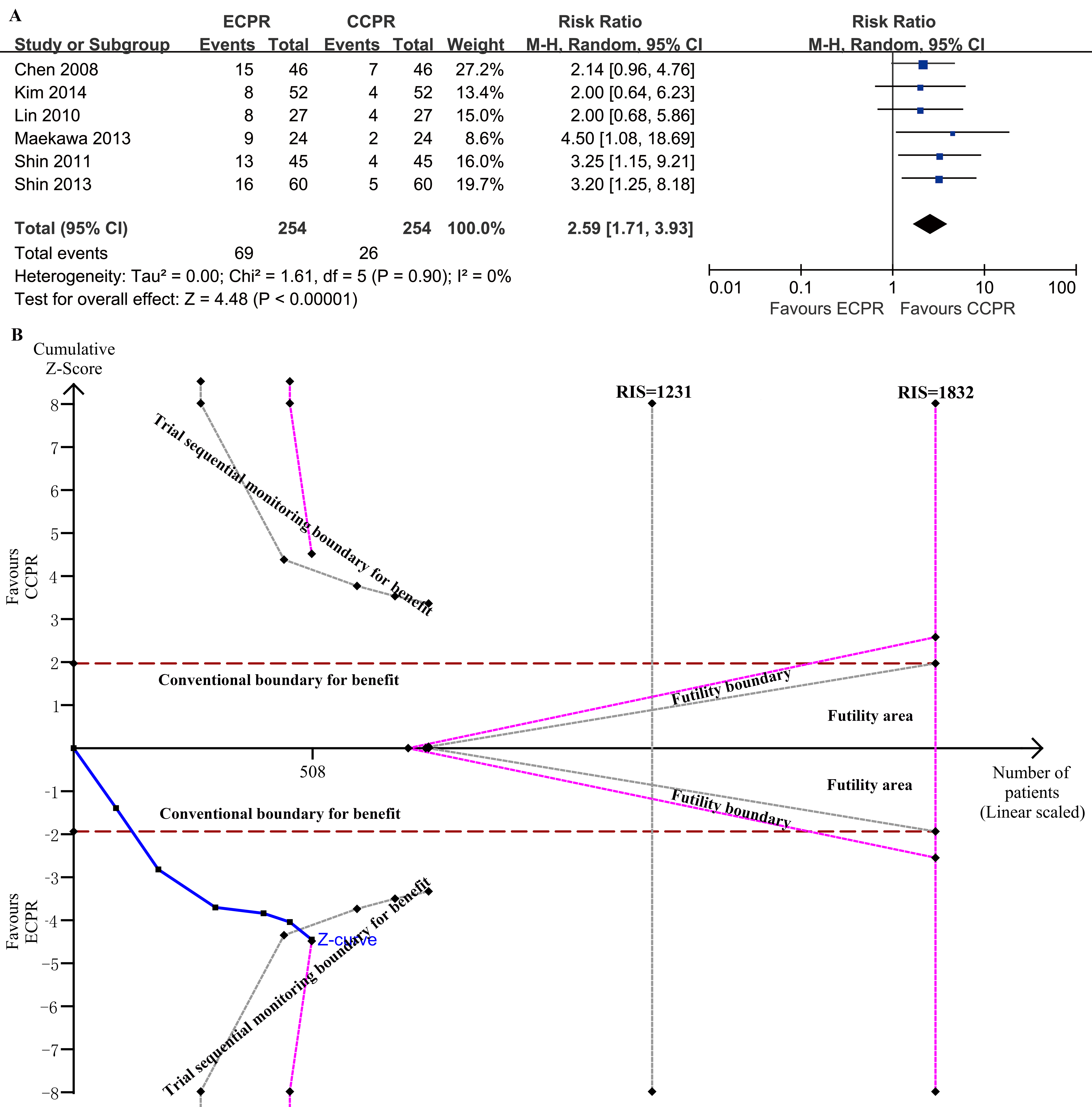

Supplement: Supplementary 1 — Supplementary Table S1: Inclusion criteria of study population and indication of extracorporeal cardiopulmonary resuscitation (ECPR). [file 6414673.f1.tif]

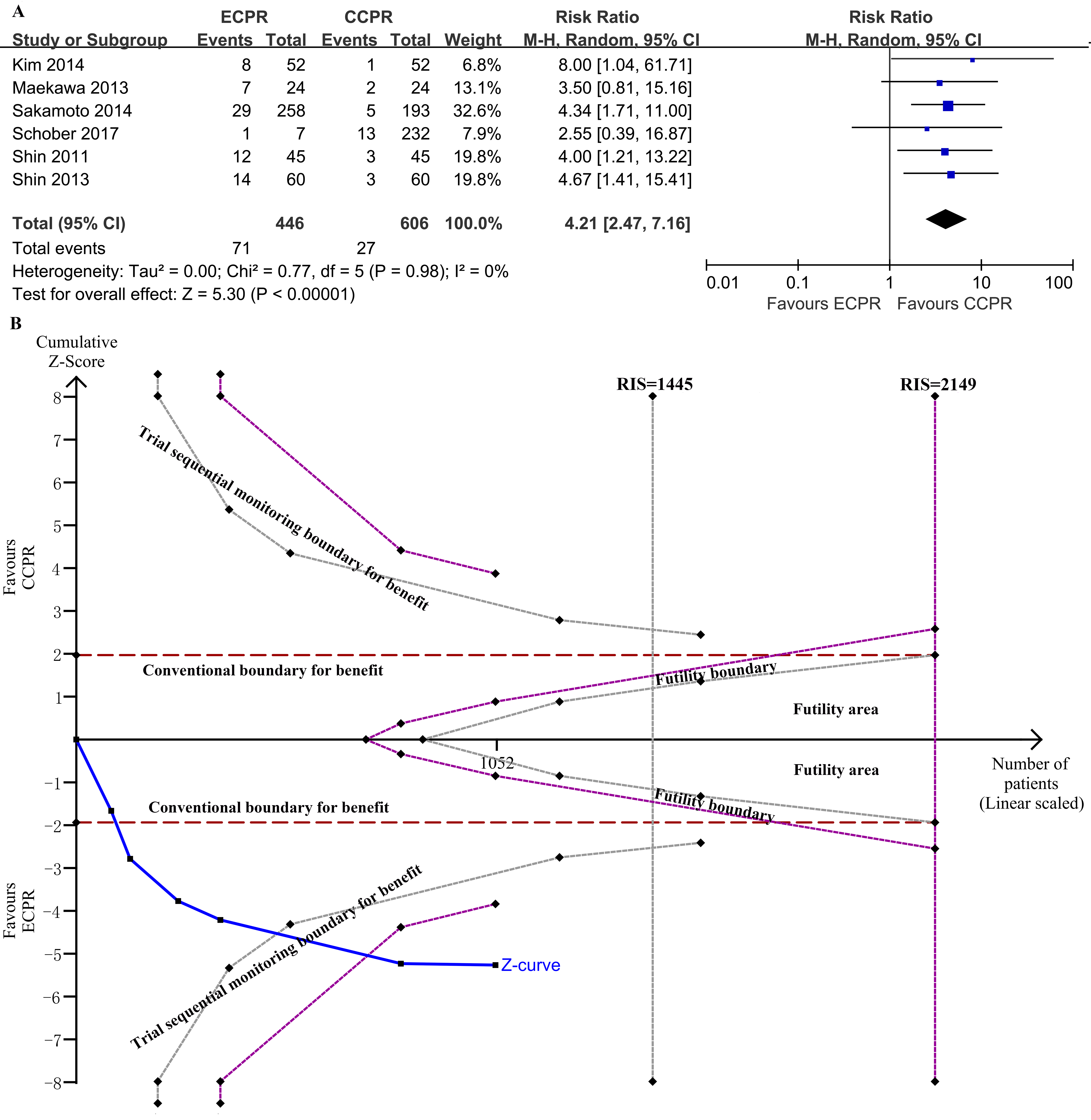

Supplement: Supplementary 2 — Supplementary Table S2: Assessment of study quality using a modified version of the Newcastle-Ottawa Quality Assessment Scale for Cohort studies. [file 6414673.f2.tif]

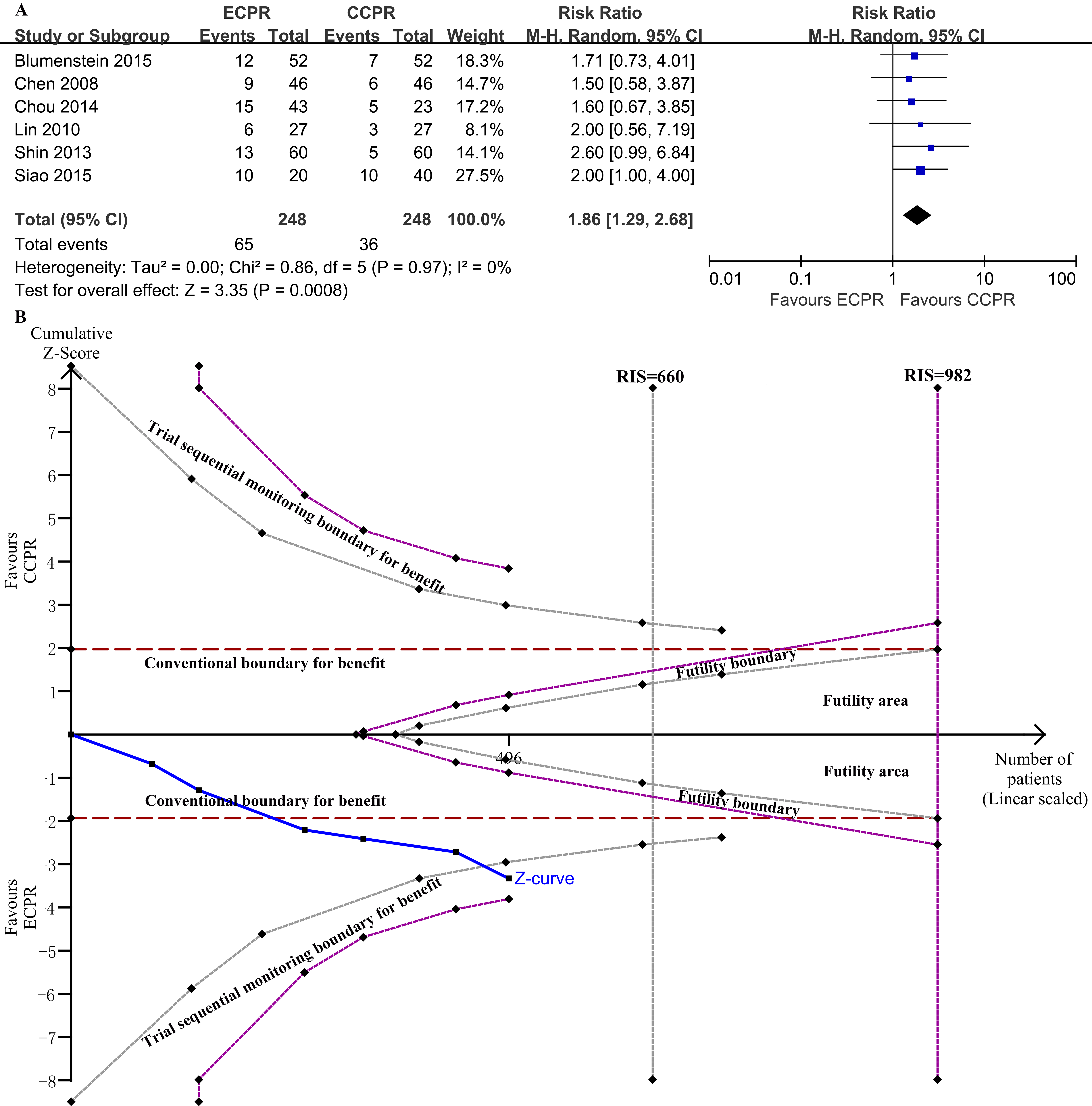

Supplement: Supplementary 3 — Supplementary Table S3: Summary of findings table. [file 6414673.f3.tif]

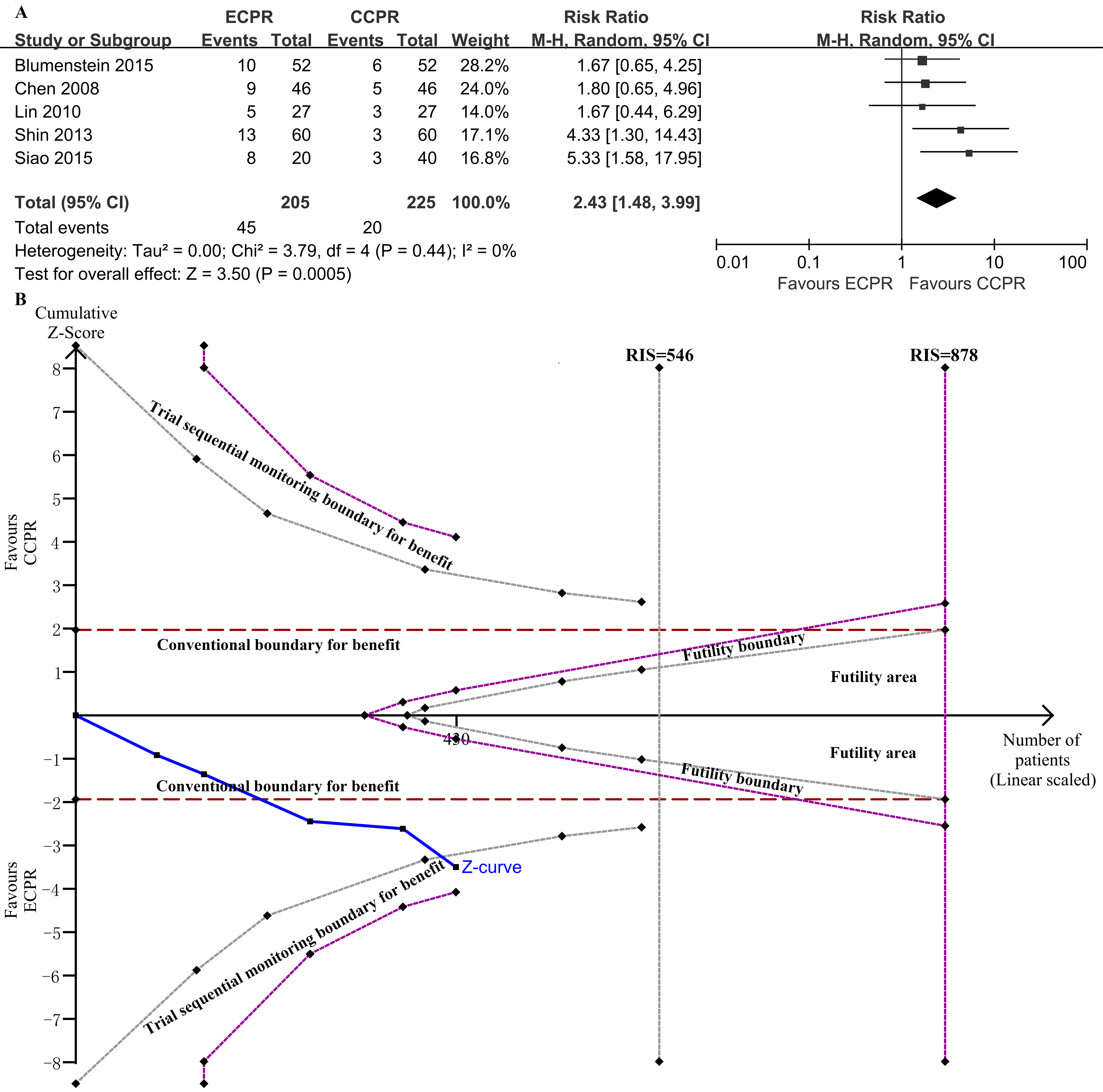

Supplement: Supplementary 4 — Supplementary Figure S1: (A) Forest plot of studies reporting 3-6 months' survival outcome. (B) Random-effect model of trial sequential analysis for 3-6 months' survival outcome. Type 1 error is =5%; a diversity-adjusted information size of 1231 participants calculated on the basis of a survival rate of 10.5% in the CCPR group, 20% increase in outcome, α = 5% (two sided), β = 20%, and I2 = 0%. Complete blue line represents cumulative Z-curve, which crossed conventional boundary (dashed red line) and the trial sequential monitoring boundary (dashed gray line). [file 6414673.f4.tif]

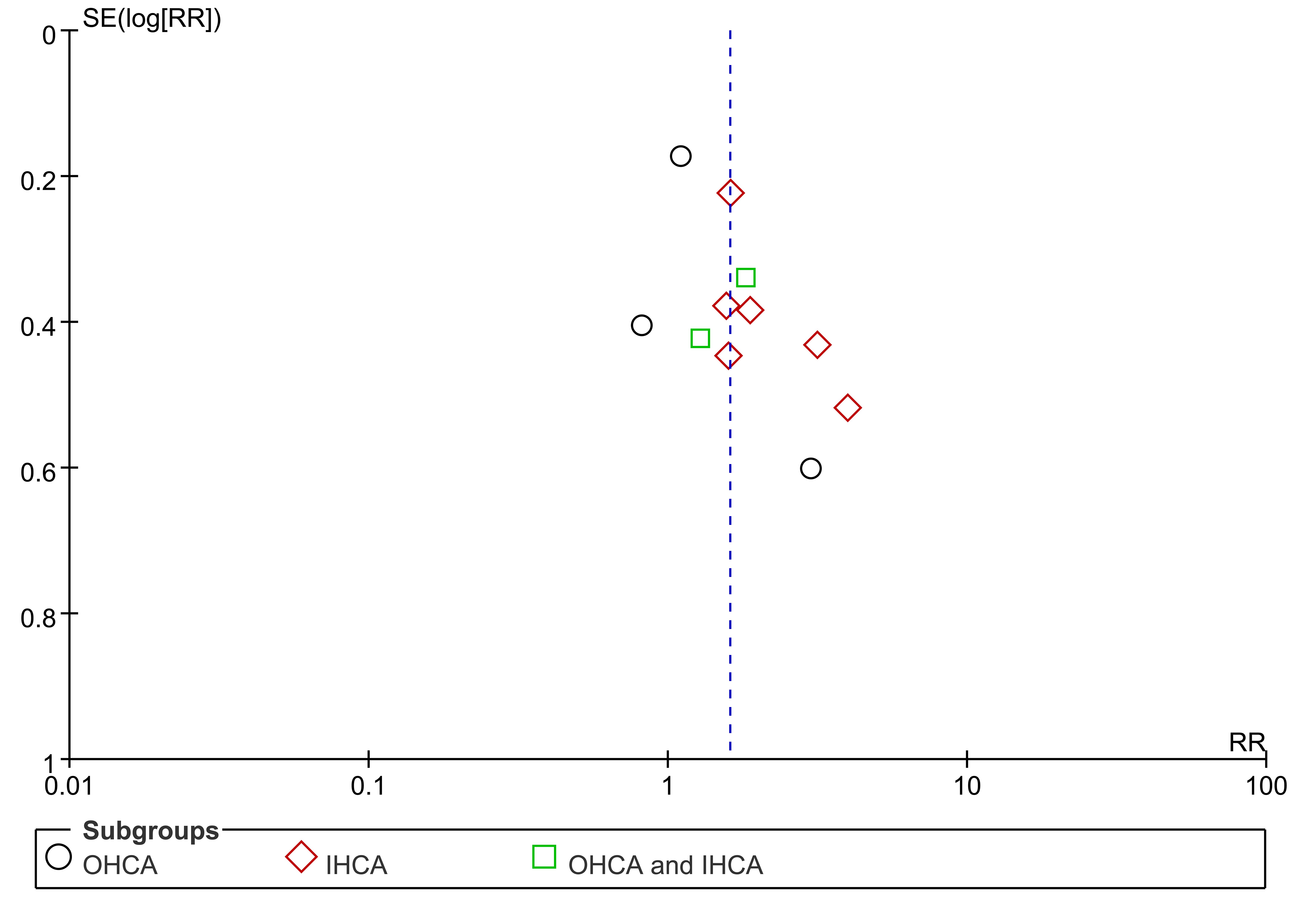

Supplement: Supplementary 5 — Supplementary Figure S2: (A) Forest plot of studies reporting 3-6 months' favorable neurologic outcome. (B) Random-effect model of trial sequential analysis for 3-6 months' favorable neurologic outcome. Type 1 error is =5%; a diversity-adjusted information size of 1445 participants calculated on the basis of a good neurologic outcome rate of 5.0% in the CCPR group, 20% increase in outcome, α = 5% (two sided), β = 20%, and I2 = 0%. Complete blue line represents cumulative Z-curve, which crossed conventional boundary (dashed red line) and the trial sequential monitoring boundary (dashed gray line). [file 6414673.f5.tif]
