# Supplementary material for: Clinical Efficacy of Extracorporeal Cardiopulmonary Resuscitation for Adults with Cardiac Arrest: Meta-Analysis with Trial Sequential Analysis
Source: Biomed Res Int. 2019 Jul 9;2019:6414673. doi: 10.1155/2019/6414673 (PMC6652040; doi:10.1155/2019/6414673)
Supplement: Supplementary 6 — Supplementary Figure S3: (A) Forest plot of studies reporting 1-year survival outcome. (B) Random-effect model of trial sequential analysis for 1-year survival outcome. Type 1 error is =5%; a diversity-adjusted information size of 660 participants calculated on the basis of a survival rate of 15.4% in the CCPR group, 20% increase in outcome, α = 5% (two sided), β = 20%, and I2 = 0%. Complete blue line represents cumulative Z-curve, which crossed conventional boundary (dashed red line) and the trial sequential monitoring boundary (dashed gray line). [file 6414673.f6.docx]

**Supplementary TableS1.*Inclusion criteria of study population and indication of extracorporeal cardiopulmonary resuscitation (ECPR)***

| Study(year) | Inclusion criteria of study population | Extracorporeal cardiopulmonary resuscitation indications | Complications |
| --- | --- | --- | --- |
| Blumenstein  (2015) | IHCA:1) witnessed | CPR > 10 min and cardiac aetiology | Cannula site complication  retroperitoneal haemorrhage and lower  limb ischaemia. |
| Chen (2008) | IHCA:1) 18~75 years, 2) CPR duration > 10min, 3) witnessed arrest of deemed cardiac origin | Prolonged CPR | Sepsis, multi-organ failure, bowel ischemia |
| Choi (2016) | OHCA:1)1) ≥18 years, 2)CPR was attempted by EMS,3) CPR was attempted by at ED,4) cardiac aetiology | Not documented |  |
| Chou(2014) | IHCA:1) >18 years, 2) sudden cardiac arrest due to AMI in the emergency department, 3) CPR duration> 10min | Activation by emergency physician, determination by cardiovascular surgeon |  |
| Kim (2014) | OHCA:1) ≥18 years, 2) no traumatic origin | 1) ≥18 years, 2) sudden arrest with presumed correctable causes, 3) witnessed arrest with or without bystander CPR or 4) no-flow time was expected to be short, even for unwitnessed arrest | bleeding at the access site, catheter insertion |
| Lee (2015) | IHCA+OHCA:1) received CPR | no ROSC after 10 minutes of CPR), 2) recurrent arrest( no ROSC more than 20min) |  |
| Lin (2010) | IHCA:1) 18~75 years, 2) CPR duration > 10min, 3) witnessed arrest of deemed cardiac origin, 4) ROSB in ECPR or ROSC in CCPR group | 1) Prolonged CPR > 10 minutes without sustained ROSC | Progressive heart failure, sepsis |
| Maekawa  (2013) | OHCA:1) ≥16 years, 2) presumed cardiac origin, 3) OHCA, 4) CPR duration > 20min after witnessed arrest | 1) no or not maintained ROSC, 2) good activities of daily life before arrest, 3)presumed cardiac origin by patient information by paramedics and rapid echocardiography, 4) emergency physician’s decision in nonelderly patient with refractory fibrillation | cannulation failure, bleeding, infection at cannulation site, leg ischemia compartment syndrome |
| Sakamoto  (2014) | OHCA:1) 20~75 years, 2) VF/VT^‡^ on initial, 3) cardiac arrest on hospital arrival, 4) within 45 min from call to hospital arrival, 5) no ROSC at least during the 15 min after hospital arrival | Not documented, 26 hospital in ECPR group, 20 hospital non ECPR group |  |
| Schober  (2017) | OHCA:1)≥18 years,2)cardiac origin,3) CPR duration>30 min | the decision was at the discretion of the treating emergency  physician |  |
| Shin (2011) | IHCA:1) 18-80 years, 2) CPR duration>10 min, 3) witnessed | 1) prolonged arrest (no ROSC after 10-20 minutes of CPR), 2) recurrent arrest(not maintained ROSC), or 3) the patient could not be expected to recover despite a short CPR duration(end-stage heart failure requiring heart transplantation, left main coronary artery occlusion, etc) | Cannulation failure, ECMO flow failure |
| Shin (2013) | IHCA:1) 20-80 years, 2) CPR duration>10 min, 3) witnessed | 1) prolonged arrest (no ROSC after 10-20 minutes of CPR), 2) recurrent arrest(not maintained ROSC), or 3) the recovery without ECMO^∥^ support was unlikely due to known severe left ventricular dysfunction or coronary artery disease | bleeding, hematoma of insertion site, vascular injury, catheter infection, limb ischemia, gastrointestinal bleeding hemolysis, stroke |
| Siao (2015) | OHCA+IHCA:1) 18-75 years, 2) CA with VF, 3) no flow time < 5min, 4) Refractory VF(resistant to at least 3 defibrillation, 3 mg Epinephrine, 300 mg amiodarone, no ROSC after CPR for more than 10min) | 1) prolonged CPR, 2) emergency physician’s decision | ECMO failure |

**Abbreviations: IHCA=In-hospital cardiac arrest, OHCA=Out-of-hospital cardiac arrest, CPR=cardiopulmonary resuscitation, ED=** **emergency department, EMS=** **emergency medical services, VF/VT= ventricular fibrillation/ventricular tachycardia, ROSC=return of spontaneous circulation, ROSB=return of spontaneous heart beat, ECMO=Extracorporeal membrane oxygenation, ECPR=Extracorporeal cardiopulmonary resuscitation, CCPR=conventional cardiopulmonary resuscitation**
