# Supplementary material for: Clinical Efficacy of Extracorporeal Cardiopulmonary Resuscitation for Adults with Cardiac Arrest: Meta-Analysis with Trial Sequential Analysis
Source: Biomed Res Int. 2019 Jul 9;2019:6414673. doi: 10.1155/2019/6414673 (PMC6652040; doi:10.1155/2019/6414673)
Supplement: Supplementary 7 — Supplementary Figure S4: (A) Forest plot of studies reporting 1-year favorable neurologic outcome. (B) Random-effect model of trial sequential analysis for 1-year favorable neurologic outcome. Type 1 error is =5%; a diversity-adjusted information size of 546 participants calculated on the basis of a good neurologic outcome rate of 9.2% in the CCPR group, 20% increase in outcome, α = 5% (two sided), β = 20%, and I2 = 0%. Complete blue line represents cumulative Z-curve, which crossed conventional boundary (dashed red line) and the trial sequential monitoring boundary (dashed gray line). [file 6414673.f7.docx]

**Supplementary TableS2.*Assessment of study quality using a modified version of the Newcastle-Ottawa Quality Assessment Scale for Cohort studies***

|  | Selection (4) | | | | Comparability (1) | | Outcome (4) | | | |
| --- | --- | --- | --- | --- | --- | --- | --- | --- | --- | --- |
| Study(year) | Study population clearly defined | >50% AMI CA with cardiac aetiology | Exposed and non-exposed cohort selected from same cohort | Definition and selection of the non-exposed cohort | Study controls for age | Study controls for CPR | Adequacy of follow up of cohorts | Follow-up long enough (>30 days) | Utstein  style | Clinical course well described* |
| Blumenstein  (2015) | **✩** |  | **✩** | **✩** | **✩** | **✩** | **✩** | **✩** |  |  |
| Chen (2008) | **✩** | **✩** | **✩** |  | **✩** | **✩** | **✩** | **✩** | **✩** |  |
| Choi (2016) | **✩** | **✩** | **✩** |  | **✩** | **✩** | **✩** |  |  |  |
| Chou (2014) | **✩** | **✩** | **✩** | **✩** |  |  | **✩** | **✩** |  |  |
| Kim (2014) | **✩** | **✩** | **✩** |  | **✩** | **✩** | **✩** | **✩** | **✩** |  |
| Lee (2015) | **✩** |  | **✩** | **✩** |  |  | **✩** | **✩** |  |  |
| Lin (2010) | **✩** | **✩** | **✩** |  | **✩** | **✩** | **✩** | **✩** | **✩** | **✩** |
| Maekawa (2013) | **✩** | **✩** | **✩** |  | **✩** | **✩** | **✩** | **✩** |  | **✩** |
| Sakamoto, (2014) | **✩** | **✩** |  | **✩** |  |  | **✩** | **✩** |  |  |
| Schober  (2017) | **✩** |  | **✩** |  |  |  | **✩** | **✩** |  |  |
| Shin (2011) | **✩** |  | **✩** | **✩** | **✩** | **✩** | **✩** | **✩** |  | **✩** |
| Shin (2013) | **✩** |  | **✩** | **✩** | **✩** | **✩** | **✩** | **✩** |  | **✩** |
| Siao (2015) | **✩** | **✩** | **✩** |  |  |  | **✩** | **✩** |  |  |

**Abbreviations: AMI= acute myocardial infarction, CA=** **cardiac arrest, CPR= cardiopulmonary resuscitation, * Revascularisation, CPR duration and complications are described.** **star (✩) was allocated to a particular item when it was adequately reported and addressed. Dashes indicate this item was not adequately reported or addressed.**
