# Supplementary material for: Clinical Efficacy of Extracorporeal Cardiopulmonary Resuscitation for Adults with Cardiac Arrest: Meta-Analysis with Trial Sequential Analysis
Source: Biomed Res Int. 2019 Jul 9;2019:6414673. doi: 10.1155/2019/6414673 (PMC6652040; doi:10.1155/2019/6414673)
Supplement: Supplementary 8 — Supplementary Figure S5: Assessment of publication bias using a funnel plot. [file 6414673.f8.docx]

**Supplementary TableS3. Summary of findings table**

| **ECPR compared to CCPR for cardiac arrest** | | | | | | |
| --- | --- | --- | --- | --- | --- | --- |
| **Patient or population:** patients with cardiac arrest **Intervention:** ECPR **Comparison:** CCPR | | | | | | |
| **Outcomes** | **Illustrative comparative risks* (95% CI)** | | **Relative effect (95% CI)** | **No of Participants (studies)** | **Quality of the evidence (GRADE)** | **Comments** |
|  | Assumed risk | Corresponding risk |  |  |  |  |
|  | **CCPR** | **ECPR** |  |  |  |  |
| **Survival outcome** Follow-up: mean 30 days | **Study population** | | **RR 1.6**  (1.25 to 2.06) | 2333 (11 studies) | ⊕⊕⊝⊝ **low**^1^ |  |
|  | **151 per 1000** | **242 per 1000** (189 to 311) |  |  |  |  |
|  | **Moderate** | |  |  |  |  |
|  | **173 per 1000** | **277 per 1000** (216 to 356) |  |  |  |  |
| **Survival outcome - OHCA** Follow-up: mean 30 days | **Study population** | | **RR 1.18**  (0.71 to 1.97) | 792 (3 studies) | ⊕⊝⊝⊝ **very low**^1^ |  |
|  | **167 per 1000** | **197 per 1000** (118 to 328) |  |  |  |  |
|  | **Moderate** | |  |  |  |  |
|  | **163 per 1000** | **192 per 1000** (116 to 321) |  |  |  |  |
| **Survival outcome - IHCA** | **Study population** | | **RR 1.9**  (1.43 to 2.52) | 1427 (6 studies) | ⊕⊕⊝⊝ **low**^1^ |  |
|  | **138 per 1000** | **263 per 1000** (198 to 348) |  |  |  |  |
|  | **Moderate** | |  |  |  |  |
|  | **155 per 1000** | **294 per 1000**(222 to 391) |  |  |  |  |
|  |  | |  |  |  |  |
| **Table5. continued** |  | |  |  |  |  |
| **Outcomes** | **Illustrative comparative risks* (95% CI)** | | **Relative effect (95% CI)** | **No of Participants (studies)** | **Quality of the evidence (GRADE)** | **Comments** |
|  | Assumed risk | Corresponding risk |  |  |  |  |
|  | **CCPR** | **ECPR** |  |  |  |  |
| **Favourable neurological outcome** Follow-up: mean 30 days | **Study population** | | **RR 2.69**  (1.63 to 4.46) | 1543 (7 studies) | ⊕⊝⊝⊝ **low** |  |
|  | **59 per 1000** | **158 per 1000** (96 to 262) |  |  |  |  |
|  | **Moderate** | |  |  |  |  |
|  | **75 per 1000** | **202 per 1000** (122 to 335) |  |  |  |  |
| **Favourable neurological outcome - OHCA** | **Study population** | | **RR 3.93**  (1 to 15.5) | 1197 (3 studies) | ⊕⊝⊝⊝ **very low**^1^ |  |
|  | **41 per 1000** | **160 per 1000** (41 to 631) |  |  |  |  |
|  | **Moderate** | |  |  |  |  |
|  | **19 per 1000** | **75 per 1000** (19 to 294) |  |  |  |  |
| **Favourable neurological outcome - IHCA** Follow-up: mean 30 days | **Study population** | | **RR 2.02**  (1.21 to 3.39) | 286 (3 studies) | ⊕⊕⊕⊝ **moderate**^1^ |  |
|  | **126 per 1000** | **254 per 1000** (152 to 427) |  |  |  |  |
|  | **Moderate** | |  |  |  |  |
|  | **135 per 1000** | **273 per 1000** (163 to 458) |  |  |  |  |
| **3-6 mouths survival outcome** Follow-up: 3-6 months | **Study population** | | **RR 2.65**  (1.75 to 4.02) | 508 (6 studies) | ⊕⊕⊕⊝ **moderate**^1^ |  |
|  | **102 per 1000** | **271 per 1000** (179 to 411) |  |  |  |  |
|  | **Moderate** | |  |  |  |  |
|  | **86 per 1000** | **228 per 1000** (151 to 346) |  |  |  |  |
|  |  | |  |  |  |  |
| **Table5. continued** |  | |  |  |  |  |
| **Outcomes** | **Illustrative comparative risks* (95% CI)** | | **Relative effect (95% CI)** | **No of Participants (studies)** | **Quality of the evidence (GRADE)** | **Comments** |
|  | Assumed risk | Corresponding risk |  |  |  |  |
|  | **CCPR** | **ECPR** |  |  |  |  |
| **3-6mouths favourable neurological outcome** Follow-up: 3-6 months | **Study population** | | **RR 4.38**  (2.56 to 7.49) | 1052 (6 studies) | ⊕⊕⊕⊝ **moderate**^1^ |  |
|  | **45 per 1000** | **195 per 1000** (114 to 334) |  |  |  |  |
|  | **Moderate** | |  |  |  |  |
|  | **53 per 1000** | **232 per 1000** (136 to 397) |  |  |  |  |
| **1 year survival outcome** Follow-up: mean 1 years | **Study population** | | **RR 1.87**  (1.29 to 2.69) | 496 (6 studies) | ⊕⊕⊝⊝ **low**^1^ |  |
|  | **145 per 1000** | **271 per 1000** (187 to 390) |  |  |  |  |
|  | **Moderate** | |  |  |  |  |
|  | **133 per 1000** | **249 per 1000** (172 to 358) |  |  |  |  |
| **1 year favourable neurological outcome** Follow-up: mean 1 years | **Study population** | | **RR 2.51**  (1.54 to 4.08) | 430 (5 studies) | ⊕⊕⊕⊝ **moderate**^1^ |  |
|  | **89 per 1000** | **223 per 1000** (137 to 363) |  |  |  |  |
|  | **Moderate** | |  |  |  |  |
|  | **109 per 1000** | **274 per 1000** (168 to 445) |  |  |  |  |
| *The basis for the **assumed risk** (e.g. the median control group risk across studies) is provided in footnotes. The **corresponding risk** (and its 95% confidence interval) is based on the assumed risk in the comparison group and the **relative effect** of the intervention (and its 95% CI). **CI:** Confidence interval; **RR:** Risk ratio; ECPR：Extracorporeal cardiopulmonary resuscitation ;CCPR: Conventional cardiopulmonary resuscitation, OHCA: Out-of-hospital cardiac arrest; IHCA: In-hospital cardiac arrest; | | | | | | |
| GRADE Working Group grades of evidence **High quality:** Further research is very unlikely to change our confidence in the estimate of effect.  **Moderate quality:** Further research is likely to have an important impact on our confidence in the estimate of effect and may change the estimate. **Low quality:** Further research is very likely to have an important impact on our confidence in the estimate of effect and is likely to change the estimate. **Very low quality:** We are very uncertain about the estimate. | | | | | | |
